# Supplementary figures and images for: In Vitro and In Silico Evaluation of Red Algae Laurencia obtusa Anticancer Activity
Source: Mar Drugs. 2023 May 24;21(6):318. doi: 10.3390/md21060318 (PMC10301910; doi:10.3390/md21060318)

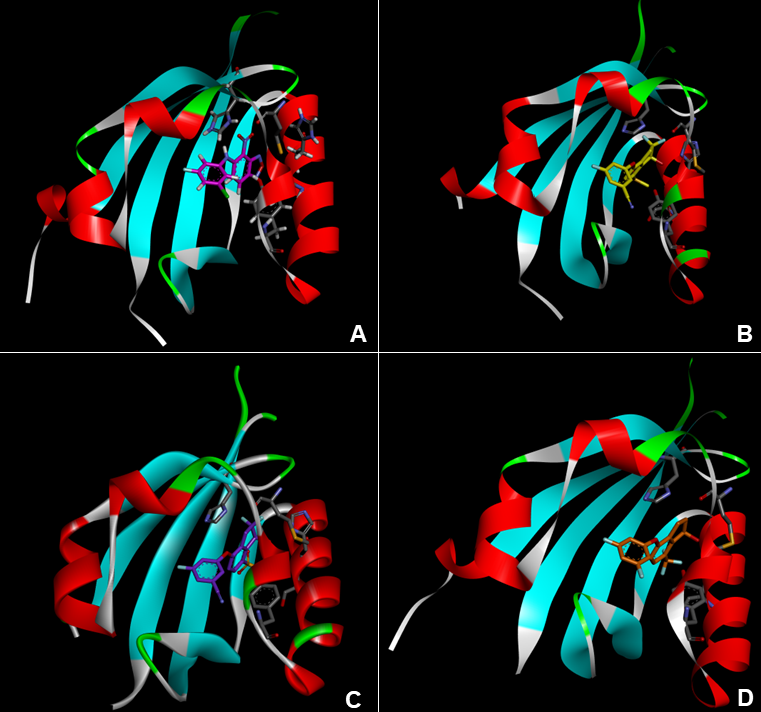

Supplement: Supplementary file 1 [file marinedrugs-21-00318-s001.zip › Figure S1. Ligands interactions in the active site of HIF-2alpha - (A) 0X3, (B) PT2385, (C) PT2399, (D) ULG.png]
